# Medical Society of the County of Albany, Semi-Monthly Meeting

**Published:** 1874-03

**Authors:** F. C. Curtis

**Affiliations:** Secretary


ART. III.—*Medical Society of the County of Albany.* Semi-Monthly Meeting, held February 18th, 1874.

Reported by F C. CURTIS, M. D. Secretary.

The Society met at the usual time and place. The President, Dr. SWINBURNE in the chair.

The following names were proposed for membership and referred to the Comitia Minora: Drs. D. COOK, C. E. BUFFINGTON, F. A. MUNSON, I. H. LENT, D. H. BARTO, WM. GEOGHEGAN, Jr., and LEWIS BALCH.

Dr. R. H. SALBIN reported a case of Nephritis occuring to a puerperal woman, and causing death in three days. The patient was 29 years of age, of tubercular tendencies, and the mother of five children. She was near the end of her pregnancy, through the course of which she had been unusually well. One evening while shaking the stove she struck herself with the handle on the knee. She was instantly taken with severe pains in the abdomen which soon went to the back and which grew steadily worse.

She was found with this continuous pain in the back, but being more severe at times she thought it the pain of labor. The os was found on examination slightly dilated and thick, hard and unyielding. She had already taken of herself eight grains of Tully's powder which she probably vomited, and the bowels had moved twice; one and a half grains of opium was administered, had chloroform by inhalation until it might have time to act, but producing little effect.

The os dilated very slowly. In about two hours the dose of opium was repeated, which allayed the restlessness of the patient, and made the pain more like that of labor—less agonizing. In about five hours the parts having dilated to admit of it, the fœtus, dead and weighing about three pounds, was delivered with the forceps. The placenta soon followed and with it three large clots, others coming away in the two or three hours succeeding. This was the only hemorrhage she had.

When seen again a few hours later she was suffering from severe after-pains, which she said was not unusual with her, and to relieve them another opium powder was given. She had passed no water since delivery and was unable to do so. Toward evening she was found very much exhausted and water was still retained. The catheter was passed but none was found in the bladder. Sweet spirits of nitre was given. She slept well during the night and was very sleepy next morning, but the urine was still suppressed.

There was a pallor of countenance, the face was puffed about the eyes, and she showed symptoms of uremic poisoning. The bladder was still found empty on using the catheter. Calomel and compound extract of colocynth of each five grains was given, to be followed in two hours with a teaspoonful of sweet spirits of nitre. At noon she had a pulse of 102, heavy respiration, with a desire to sleep. There were no pain. The bowels had not moved and the cathartic was repeated, followed in two hours by another dose and an enema of soap and water, which was not retained. At 9 P. M., there had still been no passage from the bowels although she had taken in all twenty-five grains each of calomel and colocynth extract, and half an ounce of castor oil. An enema was followed however in a few hours by a movement. No urine was passed. Next morning she felt better, the pulse was 90 and full and strong, she was restless and not so sleepy. When awake it was noted that she winked with the right eye. Her talk was disconnected, forgetting mid-way in the sentence what she was about to say. Five grains of compound extract of colocynth with one grain of elaterium was given to induce watery discharges. Toward evening she became more sleepy, so that it was almost impossible to arouse her, and about 9 P. M., she sank and died.

*Post mortem*; the bladder was found empty and dry. The kidneys were inflamed and enlarged, one of them weighing ten ounces. The uterus was contracted, and contained a dark offensive discharge. Other organs were healthy. She died of uremic poisoning.

The PRESIDENT enquired in how short a time death had been known to occur from congestion of the kidneys and suppression of urine. He had never known it to occur in less than three days. This is sometimes interesting in a medico-legal point of view. A prominent physician has asserted on the witness stand, that a person going to bed at night entirely well, may die before morning of uremic poisoning. When such an assertion is made from so high a source, it ought to be investigated.

Dr. BABCOCK remarked that this depended upon the amount of urine in the circulation. He gave the details of a case in which during four days, not more than half a pint of urine was passed, and the symptoms characteristic of congestion of the kidneys were present. The patient improved under salines, and cups with hot fomentations to the back, and recovered.

DR. CURTIS spoke concerning the part taken by the blow on the knee, in the case of Dr. Sabin, in causing the disease of the kidneys. He mentioned a case somewhat similar, which he reported to the Society last year, in which an external injury was the only cause that could be found for Brights' disease. The patient, a healthy man in advanced life and in easy circumstances, fell striking his head and baok on the pavement. Symptoms of nephritis followed within a very few days, there being at first severe pains in the head, and vomiting from the fall. There was acute nephritis followed by chronic, the disease evidently originating at the time of the fall. Death occurred in three months after the injury, and at the post mortem, the kidneys were found large, white and fatty, other organs being healthy. Dr. Sabin's case illustrates still more clearly and forcibly the effect of external injuries in causing disease of internal organs. How they produce such effects it is not easy to say, but it seems probably that it is through the nervous system, a nervous sympathy or a derangement of nerve action or supply. The exact ætiology is often an important point in a case. The writings of Brown-Sequard were alluded to.

Dr. MOSHER said it was pertinent to enquire as to the effect of the opium given in cases of acute nephritis. It is always well to make sure of the condition of the kidneys in the lying-in state before administering it, though it is generally given very indiscriminately. All anodynes seem to aggravate an existing disease of the kidneys, and the continued used of anodynes may develope it when given during the period of pregnancy. In this case a dose of Tully's powder being taken by the patient of herself might indicate that she was in the habit of keeping and using it.

Dr. LEWI said that he had never seen bad effects from opiates in either acute or chronic Brights' disease when given with discretion, symptomatically, by a careful physician.

Dr. VAN DERVEER said that he had given opium in some cases of albuminuria with pleasant results, and in others with the opposite. In one case of chronic albuminuria, convulsions came on after the exhibition of opium, and in another he had seen forty drops of laudanum by enema produce coma and death.

As to the length of time a patient lives with suppression of urine, he saw a man in May 1866, who was sick three days and recovered. Recently he was again attacked with the same, which lasted five days when he died. Although he suffered great pain but little opium was given.

Dr. J. B. STONEHOUSE, Jr., read a paper on the subject of Puerperal Insanity. This term, he said, as generally used to cover all cases of mania occurring in the pregnant and puerperal women is not strictly correct, and it is usual to divide the subject into the insanity of pregnancy, of labor including two months following the act, and of lactation.

Fifteen cases had come under his observation. Of these, eight were primiparæ, and four occurred during the second pregnancy. In four cases there were histories of heredity, and in five neuroses other than insanity were found to be hereditary. In six cases the patient had suffered from nervous disease previous to the attack, and in one an attack of chorea ceased as the insanity came on. Two cases were fatal, one from dysentery and the other from maniacal exhaustion.

As to causes of this affection, hereditary predisposition is the principal, occurring generally in the maternal side. This includes not only insanity but other neuroses. Unmarried women are more liable to it; also those reduced by excess. The general shock of labor to the nervous system is an exciting cause in one thus predisposed, and the various changes in habits and constitution following pregnancy. Albuminuria has not been found to have any connection with it.

The symptoms present no characteristic points. Melancholia is the most frequent form of insanity occurring during the period of pregnancy, and perversion of the moral element is particularly noticeable. The maniacal type is most frequent when insanity comes on during labor.

Statistics differ as to the relative frequency of its occurrence during the different periods of pregnancy, parturition and lactation.

The prognosis is most favorable when it occurs during labor, and least so when coming on during lactation.

The treatment differs little from that of non-puerperal insanity. Narcotics are to be given if warm baths and stimulants fail to quiet the patient.

A number of cases occuring in the different periods were given in detail.

Dr. Bailey said that he had met with only two cases of this affection. In both death had followed within a year from phthisis. There was a family history of this in one of them.

Dr. Mosher spoke of the large doses in which it had become the custom to administer sedatives in the insane asylums, hyoscyamus and chloral being given in combination, the latter to prolong the effects of the former. He did not consider this good practice.

Dr. Davis said that he would divide insanity into three varieties, that due to nervous irritation, deranged circulation and disorganization. In his observation puerperal insanity has been that of nervous irritation. The brain sympathizes with the uterus through the whole of gestation, and this is more marked in some than in others.

